# Effect of Microwave Maceration and SO_2_ Free Vinification on Volatile Composition of Red Wines

**DOI:** 10.3390/foods10061164

**Published:** 2021-05-22

**Authors:** Raquel Muñoz García, Rodrigo Oliver Simancas, María Consuelo Díaz-Maroto, María Elena Alañón Pardo, María Soledad Pérez-Coello

**Affiliations:** 1 Area of Food Technology, Faculty of Chemical Sciences and Technologies, Regional Institute for Applied Scientific Research (IRICA), University of Castilla-La Mancha, Avda. Camilo José Cela 10, 13071 Ciudad Real, Spain; raquel.munoz@uclm.es (R.M.G.); rodrigo.oliver@uclm.es (R.O.S.); soledad.perez@uclm.es (M.S.P.-C.); 2 Area of Food Technology, Higher Technical School of Agronomic Engineering, University of Castilla-La Mancha, Ronda de Calatrava 7, 13071 Ciudad Real, Spain; mariaelena.alanon@uclm.es

**Keywords:** microwave maceration, red wine, volatile compounds, aroma

## Abstract

This study evaluates the effect of microwave treatment in grape maceration at laboratory scale on the content of free and glycosidically bound varietal compounds of must and wines and on the overall aroma of wines produced with and without SO_2_. The volatile compounds were extracted by solid phase extraction and analyzed by gas chromatography–mass spectrometry, carrying out a sensory evaluation of wines by quantitative descriptive analysis. Microwave treatment significantly increased the free and bound fraction of most varietal compounds in the must. Wines from microwave maceration showed faster fermentation kinetics and shorter lag phase, resulting in an increase in some volatile compounds of sensory relevance. The absence of SO_2_ caused a decrease in concentration of some volatile compounds, mainly fatty acids and esters. The sensory assessment of wines from microwave treatment was higher than the control wine, especially in wines without SO_2_, which had higher scores in the “red berry” and “floral” odor attributes and a more intense aroma. This indicates that the pre-fermentative treatment of grapes with microwaves could be used to increase the wine aroma and to reduce the occurrence of SO_2_.

## 1. Introduction

For decades, the wine industry has used various technological innovations to improve the quality of its wines, but also to achieve environmentally sustainable processes that reduce the use of potentially hazardous products for health. In this sense, emerging technologies based on the use of ultrasound, microwave, or electric fields have been revealed as clean technologies that thanks to their mechanical effect on cells achieve greater performance in the extraction of compounds during maceration processes (phenolic, volatile, bioactive compounds...) [1]. These techniques can also help reduce the presence of spoilage or pathogen microorganisms, avoiding the excessive use of antiseptic agents such as SO_2_, and obtaining higher quality and healthier wines [2,3].

The aroma of wine is one of the attributes most appreciated by consumers, this aroma is formed by numerous compounds that come in part from grapes (varietal compounds) and are influenced by grape variety and agro-climatic factors. Many of the varietal compounds are found in the grape skin, either in free form as directly contributing to wine aroma or as non-volatile precursors, which release odorous molecules through enzymatic or chemical hydrolysis reactions [4,5]. Yeast metabolism also generates many volatile compounds during the alcoholic fermentation, which will be affected by the type of yeast, as well as fermentation conditions, while other aromas develop during the aging process.

Several techniques have been used to promote the extraction of compounds from grape skin during the maceration process such as the use of pectolytic enzymes, cryomaceration, or thermovinification [1,6,7]. More recently, novel techniques such as ultrasound or pulsed electric fields have been applied to red winemaking to increase the extraction of phenolic compounds and to reduce maceration and aging times [8,9,10,11]. However, its effect on the fraction of volatile compounds has been less studied. Cryomaceration has been described as promoting the extraction of varietal compounds in grapes [12]. Meanwhile, thermal processes such as thermovinification can affect the degradation of volatile compounds in the wine or promote its extraction, depending on the conditions used [13,14]. The effect of sonication during grape maceration can lead to greater extraction of the free and bound fraction of the aroma and an increase in the aroma of the final wine [15,16], although some authors describe the formation of off-flavor or do not notice changes in the aroma of wine due to sonication [17].

Microwave technology (MW) has been applied in the food industry to reduce processing times and food preservation [1]. Microwaves are non-ionizing electromagnetic waves with frequencies between 300 MHz and 300 GHz, although the frequency range for industrial use is smaller (0.9–2.45 GHz). Microwaves have a double effect on matrices: molecular movement by the migration of ions and rotation of molecules with permanent dipoles formation. The resistance offered to ion migration and the realignment of the dipoles generates friction forces that originate heating, without altering their molecular structures unless the temperature is too high [18].

This thermal energy can disrupt solute bonds with the matrix, favoring its release and helping the extraction process. If the temperature is high, a explosion of grape cells can occur by releasing its contents into the liquid phase. Microwave-assisted extraction (MAE) has been used for the extraction of analytes (volatile compounds, bioactive phenolics) in plants and wines, achieving better yields than conventional processes [19,20,21].

MW technology has been applied to reduce winemaking time by achieving higher overall wine quality [22,23]. In wines from Pinot Noir and Merlot varieties, grape maceration applying microwave succeeded in increasing the amount of total phenolic compounds, anthocyanins, and tannins of wine by shortening maceration times [24,25,26]. These authors observed an increase in the floral and fruity aroma in MW-treated wines, although volatile compounds were not analyzed. However, Tartian et al. [27] observed that microwave maceration was less effective in the extraction of volatile compounds of sensory relevance to cryomaceration or traditional maceration.

Microwave pulses have also been proposed as a method to accelerate the aging of wine as well as to favor the transfer of wood compounds in treatments with oak chips [10].

On the other hand, radiation with microwaves helps to preserve food, reducing the presence of spoilage or pathogen microorganisms [28]. Based on this fact, microwave treatment has been proposed in the oenological industry for the sanitation of reused oak barrels, achieving a reduction in the population of Brettanomyces and lactic bacteria [29]. Carew et al. [24,25] proposed the use of high-power microwaves in grape crushing inoculating with a starter culture without SO_2_ addition, seeing that fermentation was developing correctly, obtaining faster fermentation kinetics and higher yield.

The objective of this work was to study the effect of the microwave treatment at laboratory scale with temperature control during the pre-fermentative maceration of Cabernet Sauvignon grapes, on the extraction of the free and glycosidically bound fraction of volatile compounds and on the overall aroma of wines, produced with and without SO_2_ addition, with a view to its possible reduction in vinification.

## 2. Materials and Methods

### 2.1. Microwave Treatment and Microvinification

Cabernet Saugvinon red grapes were harvested at the optimal state of maturity during the 2019 harvest at the Institute of Vine and Wine of Castilla-La Mancha (IVICAM, Tomelloso, Ciudad Real, Spain). They were immediately transported to the laboratory for their processing. The chemical composition of the must was 24.9°Brix, 3.81 g/L of total acidity, and 3.26 of pH.

Grapes (150 kg) were destemmed and crushed and then divided into three batches. Two batches were sulphited at this time with 50 mg/L SO_2_, and the third batch was microwave-treated without SO_2_. One batch with SO_2_ (“C”) was not submitted to any microwave treatment and was used as control wine. This sample was immediately moved to a chamber at 22 °C (±2 °C). The other two batches (“MW”) were microwave macerated during 12 min at 700 W using a domestic LG MJ3965ACS microwave oven (LG electronics, Madrid, Spain). The samples were processed in batches of 2 kg. The temperature on the batch prior to the treatment was 14 °C, which was raised up to 40 °C when it finished. To avoid an increment up to 40 °C, the microwave treatment was realized at 3 intervals of 4 min each up, to a total of 12 min. At the end of each time interval, the batches were stirred, and their temperatures were evaluated using a solid stem thermometer. All treatments were executed by triplicate. Then, microwave treatment batches were moved to the 22 °C (±2 °C) chamber for sampling, yeast inoculation, and fermentation.

Alcoholic fermentation–maceration was realized in 10 L glass containers that were filled with 6 kg of sample (3.6 kg of must and 2.4 kg of skins (in order to not filled more than 60 %) using *Saccharomyces cerevisiae* (CECT no. 10835) as starter culture, at 22 °C (±2 °C). They were punched down twice every day. The evolution of the fermentation was controlled by weight loss, and it was considered finished when the container weight remained stable. The final of the fermentation was confirmed by density and glucose and fructose analysis of wines. Finally, wines were decanted, filtered, and bottled. Only wines with SO_2_ were adjusted to 25 mg/L of free SO_2_ before bottling when it was necessary.

### 2.2. Solid Phase Extraction

Solid phase extraction (SPE) was used for the isolation of the free and glycosidically bound volatile compounds of musts and wines. Before SPE, musts were centrifuged at 4 °C (10,000 rpm, 10 min) (Avanti Centrifuge J26-XP, Beckman Coulter, CA, USA) and filtrated through a 1.2 μm glass fiber membrane (Fisherbrand, Thermo Fisher Scientific, Inc., Waltham, MA, USA), while wines did not need prior preparation. All extractions were carried out in duplicate according to the method described by Oliver Simancas et al. [16].

#### 2.2.1. Free Volatile Compounds

First, the SPE cartridges (500 mg styrene-divinylbenzene, Lichrolut EN Merck, KGaA, Darmstadt, Germany) were conditioned with 10 mL of dichloromethane, 5 mL of methanol, and 10 mL ethanol:water (10:90, *v*/*v*). Then, 100 mL of sample together with 40 μL of the internal standard (4-nonanol, 1 g/L) were passed through the cartridge. Non-volatile hydrophilic compounds were washed out of the cartridges with 50 mL of bidistilled water (Milli Q Plus, Merck KGaA, Darmstadt, Germany), and the free volatile compounds were eluted with 10 mL of dichloromethane. The extracts were concentrated to an approximate volume of 200 μL under nitrogen and stored at −20 °C until their analysis.

#### 2.2.2. Glycosidically Bound Volatile Compounds

Once the free volatile compounds were removed, glycosidically bound fraction was eluted from the same cartridge with 25 mL of ethyl acetate:methanol (90:10, *v*/*v*). Extracts were evaporated under vacuum (55 °C, 290 mBar) to almost dryness (≈1 mL), achieving complete dryness under a gentle stream of nitrogen. To recover the dry extract distributed in the flask walls, it was re-dissolved with 1 mL of methanol and evaporated again with nitrogen until dryness. The enzymatic hydrolysis was performed by adding 0.20 μL/mL of a commercial enzyme “Trenolin Bouquet PLUS” (ERBSLÖH, Geisenheim, Germany) in 2 mL of phosphate-citrate buffer (0.1–0.2 M; pH = 5),and incubating at 40 °C for 16 h. Then, 25 mL of synthetic wine (12% ethanol, pH = 3.5) were added to the flask, and the released volatile compounds were recovered by SPE, using 200 mg styrene-divinylbenzene cartridges (Lichrolut EN Merck, KGaA, Darmstadt, Germany) previously conditioned (5 mL of dichloromethane, 2.5 mL of methanol, and 5 mL of 10 % ethanol:water). Hydrophilic compounds were removed from the cartridges with 25 mL of bidistilled water (Milli Q Plus, Merck KGaA, Darmstadt, Germany), and released volatile compounds were eluted with 5 mL of dichloromethane. The extracts were concentrated to an approximate volume of 200 μL under nitrogen and stored at −20 °C until their analysis.

### 2.3. Gas Chromatography-Mass Spectrometry

Extracts (1 µL) were analyzed in an Agilent 6890 GC System coupled to an Agilent 5973 inert Mass Selective Detector and equipped with a DB-WAX column (60 m × 0.25 mm × 0.25 µm) (Agilent Technologies, Inc.), using helium as the carrier gas (1 mL/min). The oven temperature program started at 70 °C for 5 min; then, it was raised up first at 1 °C/min to 90 °C (10 min) and then at 2 °C/min to 210 °C, which was maintained for 40 min. The injector temperature was 250 °C. The MS conditions were: electron impact mode (70 eV), ion source temperature of 230 °C, and scanning from 45 to 550 a.m.u.

Identification of the volatile compounds was carried out by comparison with standards from Sigma-Aldrich (Tres Cantos, Madrid, Spain). Compounds for which it was not possible to find volatile references were tentatively identified by comparison of their mass spectra with those from different mass spectrum libraries (Wiley G 1035 A, NBS75K and NIST14). Quantitative analysis (µg/L) was performed considering the response factors of the different volatile compounds, except for those whose commercial standards were not available, which were quantified by means of the response factors of compounds with similar chemical structures: geranial was quantified as *trans*-geraniol; 3-oxo-α-ionol, 3-hydroxy-7,8-dihydro-β-ionol, and 6,7-dehydro-7,8-dihydro-3-oxo-ionol were quantified as α-ionol; 4-methyl-1-pentanol was quantified as 3-methyl-1-pentanol; isovaleric acid was quantified as propanoic acid; isobutyric acid was quantified as butanoic acid; ethyl 3-hydroxyhexanoate was quantified as ethyl hexanoate; and methyl 2-hydroxy-4-methylpentanoate and ethyl 4-hydroxybutyrate were quantified as ethyl 3-hydroxybutyrate.

### 2.4. Descriptive Sensory Analysis

Wines were tasted, in duplicate, three months after bottling, by eight judges with high experience in wine sensory analysis, two men and six women, aged between 23 and 62, belonging to the staff of the Food Science and Technology Area of the University of Castilla-La Mancha. Evaluation took place in a standard sensory analysis chamber (ISO 8589:2007) equipped with separate booths. While wines were presented in standard wine-tasting glasses (ISO 3591:1997). Samples were sniffed and tasted, and the judges generated sensory terms individually, agreeing on the following olfactory descriptors: floral, green, red berry, prune, and odor intensity. A 10 cm unstructured scale was used to measure each attribute, indicating in the left extreme “attribute not perceptible” and in the right extreme “attribute strongly perceptible”.

### 2.5. Statistical Analysis

Student–Newman–Keuls’s test was used to find significant differences between the volatile compounds of the samples, while ANOVA analysis was applied to sensory data. Both analyses were carried out with the IBM SPSS statistical package, version 24.0 (IBM, Madrid, Spain).

## 3. Results and Discussion

### 3.1. Effect of Microwave Treatment on Volatile Compounds of Musts

**Figure 1:**
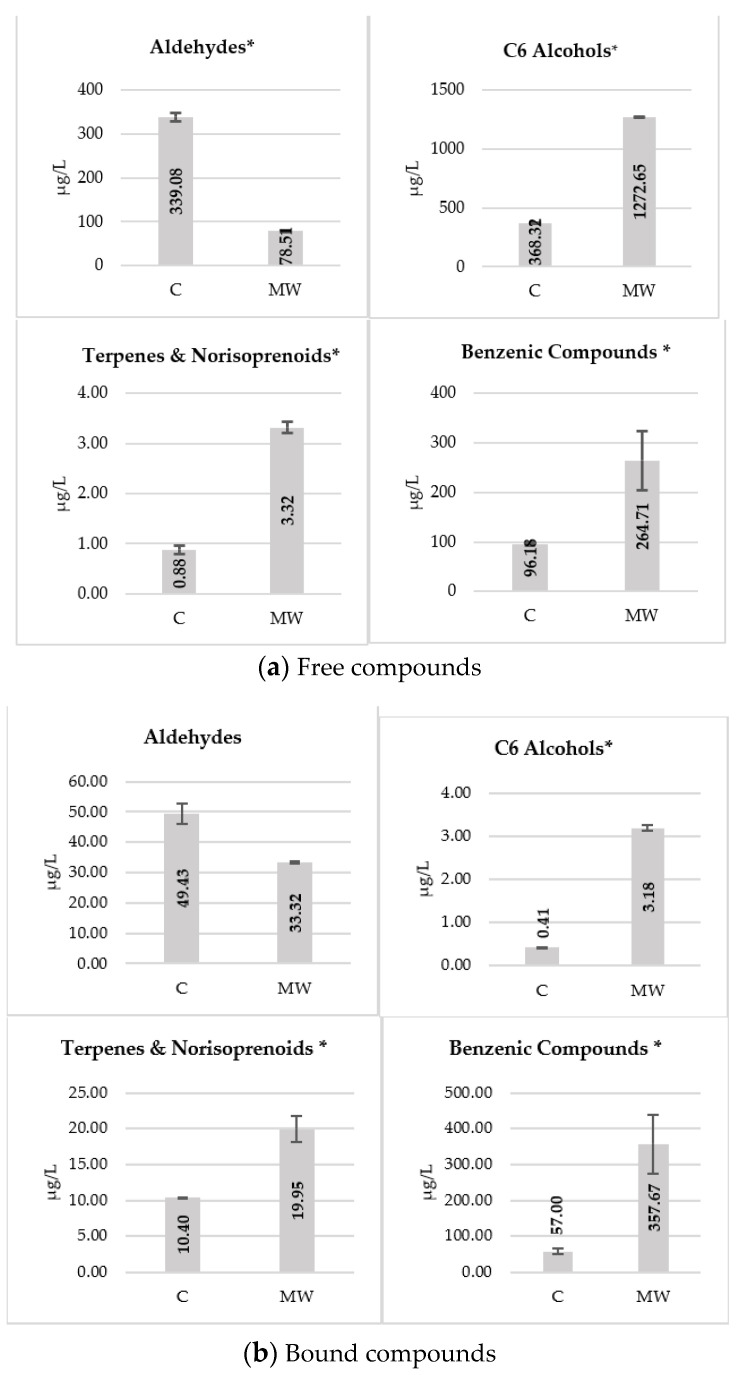
Mean concentrations (μg/L) of the main groups of free (**a**) and bound compounds (**b**) in control must (C) and must from microwave-treated grapes (MW). *: denote significant differences according to Student’s t-test (*p* ≤ 0.05).

Figure 1a,b shows the main groups of varietal volatile compounds quantified in the free and bound fraction of the musts, respectively. Aldehydes and C6 alcohols were the majority groups. These compounds are formed by the degradation of lipids from the grape skin in the presence of oxygen, mainly in pre-fermentation processes.

Hexanal was the majority aldehyde in the control must followed by trans-2-hexenal (Table S1 Supplementary Material). The aldehydes are characterized by their pleasant aromas (sweet, orange) and low olfactory detection thresholds but, similar to other low molecular weight aldehydes, they are transformed during pre-fermentative and fermentative processes into their corresponding alcohols [4].

It should be noted that free aldehydes had much lower concentrations in microwave-treated musts (Figure 1a), whereas the opposite occurred with the corresponding alcohols. Among these, 1-hexanol and *cis*-2-hexen-1-ol were the majority compounds in musts treated with microwaves. This suggests that microwave treatment significantly increasing their extraction from grape skin, especially in the case of alcohols.

The bound fraction of the aldehydes and C6 alcohols was much lower than the free fraction, which has been previously described [4], and the effect of microwave maceration was not significant.

Terpenes and norisoprenoids are mainly in their bound form and may also be affected by intensive maceration techniques [30]. In fact, although they were found in small amounts, their concentration in both free and bound form increased in the musts treated with MW.

Benzenic compounds had similar amounts in their free and bound form, with a very positive effect of microwave treatment observed in the extraction of both fractions, which greatly increased in their bound form. This effect was also observed in grape ultrasonic maceration [16]. The major compounds were benzyl alcohol and 2-phenylethanol, as well as others such as 4-vinylguaiacol or vanillin (Tables S1 and S2, Supplementary Material), whose quantities in wines will be increased by the metabolism of yeasts and may reach concentrations of sensory significance.

No previous work has been found on the effect of using MW in grape maceration on volatile compounds of musts. However, further extraction of phenolic compounds and amino acids in high-powered MW maceration has been described, by proposing this technique for obtaining Pinot Noir wines with greater color reducing maceration times [23,24].

Other authors have observed an increase in the free and bound fraction of must volatiles applying various techniques in the grape maceration step as ultrasounds and pulsed electric fields [8,16,31].

Likewise, the effect of microwaves on skin cells has been able to facilitate the release of volatile compounds free and glycosidically bound into the liquid phase. In any case, the use of maceration with MW would increase the aromatic potential of grape musts.

### 3.2. Effect of Microwave Treatment on Volatile Compounds of Wines Fermented with and without SO_2_.

The grape must from pre-fermentative maceration with MW and the control must were vinified in the traditional way with SO_2_ addition. However, due to the inhibitory effect of microwaves on the native microbial population described by some authors [24,25], parallel fermentation of the microwave-treated must without SO_2_ was carried out. Table 1 and Table 2 show the results of the free and glycosidically bound fraction of the varietal compounds quantified in wines.

C6 alcohols are one of the main components of the free fraction, with 1-hexanol as the major compound (Table 1). During the winemaking, C6 alcohols can suffer more modifications, especially in the maceration phase in red wines, in which the release of enzymes and precursors from the grape skin continues.

Wines from MW-treated musts presented higher quantities in total C6 alcohols, especially 1-hexanol, indicating their release from the grape skin cells during fermentation. However, a smaller increase was observed in those fermented wines without SO_2_, which was possibly because oxidation reactions were favored in these conditions. The higher extraction of C6 alcohols can cause green and herbaceous aromas in wines, so it is not desirable to exceed its olfactory thresholds in wines [4].

The bound fraction of C6 alcohols was minority as in the must, and the effect of microwave maceration was scarce (Table 2). Some authors have also observed an increase in C6 alcohols in red wines from grapes undergoing sonication treatment during grape maceration [15,16].

Terpenes and norisoprenoids are compounds of sensorial relevance as they can contribute in an individual or synergistic way to the overall aroma of wines. The free fraction of these compounds was overall quantitatively important in all wines (Table 1), but an increase in wines from microwave maceration was observed, although the absence of SO_2_ also decreased its concentration, except in the case of linalool. It has been described that many terpenes are very sensitive to oxidative processes, so the absence of SO_2_ has been able to cause a loss of protection against oxidation [32].

Monoterpenes such as linalool, α-terpineol, and geraniol have floral or fruity aromas and low perception thresholds. Geranial was the majority terpene in wines from microwave maceration; this compound could have been formed by geraniol oxidation.

The most relevant compound within the norisoprenoids was the β-damascenone (floral, sweet, and honey-like aroma), which appeared in all samples at concentrations above its olfactory detection threshold (0.05 μg/L) [33]. Control wines had greater concentration, showing a possible degradation by MW treatment.

The glycosidically bound fraction of terpenes and norisoprenoids present in the wines was very small compared to the free fraction, which assumes that during the fermentation process, an important release of its precursors has occurred thanks to the glycosidic activity of yeasts [34,35]. MW treatment showed only a slight increase of this fraction in samples.

The effect of different maceration techniques on the extraction of terpenes and norisoprenoids has been studied by several authors, although the results depend on the conditions of the treatment applied. Tartian et al. [27] obtained higher amounts of monoterpenes in wines from cryomaceration than in those treated with ultrasound or microwave. Must thermovinification negatively affected the terpene content in wines [14]. In Monastrell wines from sonicated grapes, no changes in terpenes or norisoprenoids were observed [11,17].

Finally, in the group of benzenic compounds, we found quantitatively important compounds in both free and bound fractions (Table 1 and Table 2). It should be noted that although they come from grapes, some of them can also be formed by the metabolism of yeasts, such as 4-vinylguaiacol or benzyl alcohol. MW-treated wines showed no significant differences in total benzenic compounds from control wines in both free and bound fraction.

Some sensory-relevant compounds such as guaiacol (smoky, sweet, medicinal aroma) and 4-vinylguaiacol (spicy aroma) were found in amounts above their perception thresholds in samples. Vanillin and its derivatives can jointly influence the vanilla aroma of wines. However, they all had lower concentrations in wines from microwave maceration and were unaffected by the absence of SO_2_.

Benzenic compounds increased considerably in musts treated with microwaves, both in the free and bound fractions. However, in the wines, the differences due to MW treatment were smaller. During the red wine fermentation, grape skins’ extraction of compounds was also possible slowly in the control wines, while MW treatment caused a much faster release of compounds at the beginning of fermentation. In the final wine, the quantities are balanced. Carew et al. [23] observed that the use of MW can favor the rapid release of phenolic compounds when shorter maceration processes are required in early press of Pinot Noir wines.

Similarly, the use of ultrasounds in red grape maceration was not effective in the extraction of benzenic compounds in red wines of different varieties [16,17], while grapes maceration with pulsed electric fields reduced the concentration of volatile phenols in white wines [8]. These authors justify this fact by a decrease of the hydroxycynamic acids precursors in the must via oxidation or because the enzymes involved may be affected.

### 3.3. Effect of Microwave Treatment on Volatile Compounds Formed during Alcoholic Fermentation with and without SO_2_.

Volatile compounds formed during alcoholic fermentation form the basis of the wine aroma, especially in young wines from neutral grapes [36]. Grape maceration with MW has been shown to increase the amounts of phenolic compounds, amino acids, and other nutrients that can be used by yeasts as precursors of volatile compounds [24,25]. Likewise, the absence of SO_2_ can influence the selection of yeasts that carry out fermentation by favoring the development of native yeasts and influencing the implantation of the starter, which could modify the profile of metabolites formed during fermentation.

**Figure 2:**
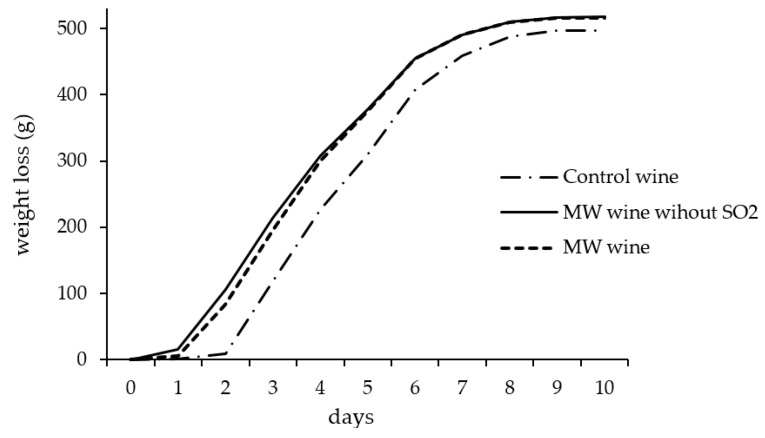
Alcoholic fermentation kinetics indicated by means weight loss in control wine and wines from microwave maceration elaborated with and without SO_2_. “Control wine”: no treatment applied; “MW wine”: microwave treatment at 700 W, 12 min of grape crushed; “MW wine without SO_2_”: microwave treatment at 700 W, 12 min of grape crushed and elaborated without SO_2_.

Figure 2 shows the monitoring of wines fermentation, representing the average weight loss value of triplicates. As can be seen, the lag phase was longer in the case of control wine, since the weight loss of the flasks started later, while microwave-treated wines began fermentation more quickly. Wines from MW maceration also showed faster fermentation kinetics and higher fermentation yield, which was slightly higher in the absence of SO_2_; this may be due to the lack of inhibition of yeasts due to SO_2_. This effect has also been observed in wines from Pinot Noir elaborated with MW maceration at high potency [24,25] and with other physical treatments used in grape maceration such as sonication [11]. Treatment with MW, as well as sonication, could achieve a greater extraction of compounds from the inside of the grape such as amino acids, fatty acids, and other nutrients that the yeasts could use for their growth, producing a greater quantity of metabolites and accelerating the fermentation process.

Table 3 shows the main volatile compounds produced during the alcoholic fermentation. Higher concentrations of most alcohols were identified in wines elaborated from microwave-treated grapes. This fact is justified by a greater extraction of amino acids and other volatile precursors and a more efficient fermentation due to MW treatment, which causes a greater production of metabolites by yeasts during alcoholic fermentation. The absence of SO_2_ resulted in an increase in some major alcohols such as 2-methyl-1-propanol and 2-phenylethanol, which was characterized by its aroma of roses. It has been shown that the low availability of oxygen in fermentations with SO_2_ can affect yeast metabolism by influencing the formation of alcohols and esters [37].

Fatty acids were most abundant in wines with MW maceration fermented with SO_2_, with isovaleric acid and octanoic acid being the majority in all samples. Treatment with MW could favor the extraction of precursors of these acids from grape as unsaturated long chain fatty acids. Fatty acids have no pleasant aromas (rancid, cheese, or fatty), although they are usually found in wine below their olfactory thresholds. However, they are precursors of the fatty acid esters, among which we find compounds of great sensory relevance in young wines [36].

The amount of fatty acid esters in microwave-macerated wines was significantly higher than in control wines, although the absence of SO_2_ caused the decrease of some of them (ethyl octanoate, ethyl decanoate), which is consistent with its content of fatty acids since they share the metabolic pathway from Acetyl-CoA. Short-chain fatty acid ethyl esters with low olfactory thresholds offer wines fruity notes. Ethyl butyrate (with strawberries, apple aroma) and ethyl hexanoate (with fruity, green apple aroma) were found in all wines above their odor thresholds [33]. Ethyl isovalerate characterized by its apple, “sweet aroma” with a low odor threshold (3 μg/L) [38] was more abundant in MW-treated wines elaborated without SO_2_.

As for acetates, isoamyl acetate (with “banana aroma”) and 2-phenyl ethyl acetate (“roses”, “floral” aroma) were the most quantitatively and sensory important compounds. In both cases, the MW macerated wines in the presence of SO_2_ obtained the highest concentrations, although the olfactory thresholds were exceeded in all samples [33]. Garde-Cerdan and Ancin-Azpilicueta [37] did not observe differences in the amounts of esters and acetates in vinifications without SO_2_, except for the ethyl hexanoate that increased. In our case, a significant decrease was observed, which indicates the influence of oxygen availability and must composition on the production of metabolites by yeasts.

Within the hydroxyacids, the most abundant was the 4-OH-ethyl butyrate that presented the highest concentration in the control wine. However, the ethyl 2-hydroxy-4-methylpentanoate related with the blackberry aroma in red wines was the most significant due to its lower odor thresholds [39] and did not undergo modifications due to MW treatment.

Benzenic compounds include 4-vinylphenol synthesized by yeasts from the cinnamic acids of the grape, which justifies that a greater extraction of these acids increases their concentration in MW-treated wines. Due to its unpleasant odor (medicinal, pharmaceutical), it is not advisable that its concentration be increased in wines. 2-Phenylethanol and tyrosol belong to the group of major alcohols that are formed from certain precursor amino acids. A greater extraction of amino acids due to microwave treatment, as well as the greater fermentative activity observed in these wines would justify their greater presence in the wines treated with MW.

Sulfur compounds (methionol and 3(2H) dihydro 2-methyl thiophenone) were more abundant in control wines. Methionol is very sensitive to oxidation, forming methional with a lower detection threshold; however, its decrease in the absence of SO_2_ has been observed, which is probably due to less sulfur input. [40].

Lactones usually contribute pleasant aromas to the wine. Of the lactones identified, the γ-butyrolactone was the majority, being more abundant in the control wines. However, other lactones such as γ-nonalactone (coconut-like aroma) and γ-decalactone showed higher concentrations in wines with MW maceration.

In general, wines from microwave-treated grapes had higher quantities of most fermentation compounds, especially some esters, acetates, and alcohols of sensory interest. However, the absence of SO_2_ resulted in a decrease in the concentration of some of these compounds, which could be justified by changes generated by oxidative processes or due to different metabolic routes used by yeasts depending on the availability of oxygen.

The works about the use of other alternative techniques in grape maceration such as sonication showed a variable behavior on its effect in volatile fermentation compounds, depending on the grape variety and the conditions used (maceration time, power used, etc.). Lower production of acetates, increasing of total esters and alcohols, and no changes have been observed by different authors [15,16,41]. Geffroy et al. [14] observed that the heating temperature in thermovinification did not induce any changes in ethyl esters, acetates, and acids, despite the increase in yeast assimilable nitrogen (YAN) due to treatment.

### 3.4. Sensory Analysis of Control and Microwave-Treated Wine Elaborated with and without SO_2_

Changes observed in volatile composition due to microwave treatment in grape maceration and absence of SO_2_ may have sensory effects on wines. Therefore, an olfactory descriptive sensory analysis of wines was carried out. The olfactory attributes that best represented the samples selected by the panel of judges were red berry, prune, green, floral, and odor intensity.

**Figure 3:**
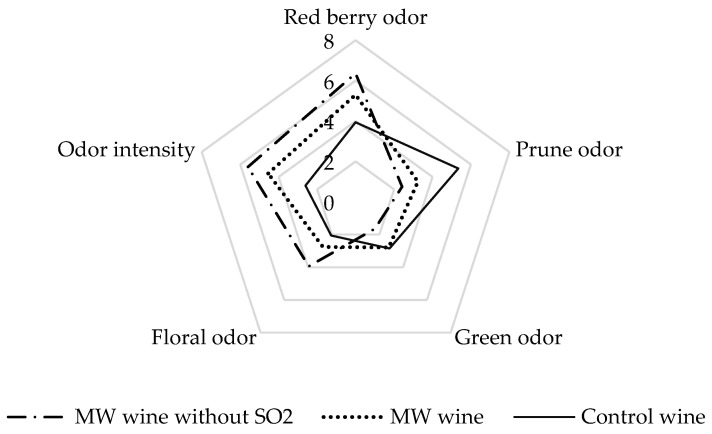
Olfactory attributes scores of control wines and wines from microwave maceration elaborated with and without SO_2_. “Control wine”: no treatment applied; “MW wine”: microwave treatment at 700 W, 12 min of grape crushed; “MW wine without SO_2_”: microwave treatment at 700 W, 12 min of grape crushed, elaborated without SO_2_.

Figure 3 shows in the form of a spider web the scores given by the tasters to these attributes in control wines and those treated with microwaves (with and without SO_2_). All attributes showed significant differences between samples according to the ANOVA test. The wines from maceration with microwave were the ones that had the highest scores in the attributes floral and red berry odor, as well as a greater intensity in the aroma. This may be related to its higher content in esters and acetates fundamentally. Table 4 shows the odor active values (OAVs) of those compounds whose concentrations in the tested wines were closer to their perception thresholds, and therefore they may have a greater sensory impact. As can be seen, the microwave-treated wine presented the highest OAVs values in all fatty acid esters with descriptors related to fruit attributes that exceeded their detection threshold. However, some berry notes have been linked in red wines to certain profiles of ethyl esters, including odorants at subthreshold concentrations [42].

Scores for floral and red berry attributes were also higher for wines made without SO_2_, which may be related to the higher OAVs in compounds such as linalool (with a OAV close to 1), ethyl isovalerate with apple, sweet aroma, and 2-phenylethanol, characterized by its rose aroma, in these wines. Other authors have described an increase in the notes related to floral and fruity aroma in wines elaborated with different SO_2_ substitutes, suggesting that the presence of SO_2_ could mask and neutralize these aromas [43,44].

Prune aroma was identified by tasters in greater quantities in control wines. This aroma has been linked to the presence of high levels of β-damascenone and γ-nonalactone [45]. The first was the compound with the highest odor active value in all samples, but the control wine presented the highest values.

The green odor attribute may be related to the presence of C6 alcohols, which were overall superior in microwave-treated wines due to their greater extraction of the grape skin. Therefore, it is logical to think that this attribute will increase in these wines, although their scores were not excessively high; in fact, none of them exceeded their olfactory threshold individually. This fact is common in wines undergoing intense maceration treatments, such as sonication or cryomaceration [16].

The effect of other grape maceration techniques on the sensory perception of wines differs according to the studies carried out. Maceration with ultrasound and thermovinification increased floral and fruity scores, obtaining greater aromatic complexity in Syrah and Monastrell wines [15,16,46]. No defect or unpleasant aromas were detected in the wines analyzed, which has been described with other intensive maceration techniques [47]. Some compounds such as guaiacol or vinylguaiacol that presented OAVs higher than 1, which is related to a spicy or medicinal aroma, were not detected.

## 4. Conclusions

The microwave treatment with medium intensity (700 W) and temperature control applied in the maceration of grapes crushed at laboratory scale increased the amounts of varietal compounds of the must in a very evident way in both the free and the glycosidically bound fractions. This increase may be due to a greater extraction of these compounds from the grape skin thanks to MW treatment.

Wines from MW maceration in the presence of SO_2_ obtained higher amounts of C6 alcohols, terpenes, and norisoprenoids in free form, while there were few changes in the bound fraction and in the benzenic compounds concentration. On the other hand, wines with MW treatment showed faster fermentation kinetics and shorter lag phase, resulting in an increase in some volatile compounds from fermentation of sensory relevance such as alcohols, esters, and acetates. The absence of sulfurous in treated MW wines resulted in changes in the concentrations of some volatile compounds, such as a decrease in some esters or an increase in linalool or 2-phenylethanol.

In addition, the wines best valued by the tasters for their greater red berry and floral odor and intensity aroma were those treated with MW, and especially those elaborated without SO_2_, which shows that treatment with MW can be very suitable to increase the aromatic potential of wines by reducing SO_2_ levels in their production.

## Figures and Tables

**Table 1:** Free volatile compound concentrations (μg/L) in control wines and those obtained from microwave-treated grapes elaborated with and without SO_2_ (*n* = 3).

Volatile Compounds	Control Wine	MW Wine	MW Wine without SO_2_
	Mean ± SD	Mean ± SD	Mean ± SD
1-hexanol	227.32 ± 48.70 ^a^	812.81 ± 33.61 ^c^	553.42 ± 76.76 ^b^
*cis*-3-hexen-1-ol	19.92 ± 2.00 ^a^	41.78 ± 2.35 ^b^	17.09 ± 1.67 ^a^
*trans*-3-hexen-1-ol	4.25 ± 0.58 ^a^	10.54 ± 0.72 ^b^	4.92 ± 0.49 ^a^
*cis*-2-hexen-1-ol	8.94 ± 1.06 ^c^	4.18 ± 1.06 ^b^	1.48 ± 0.28 ^a^
*trans*-2-hexen-1-ol	2.89 ± 0.69 ^a^	4.97 ± 0.38 ^b^	4.02 ± 0.88 ^a,b^
Σ C6 alcohols	263.32 ± 50.24 ^a^	874.28 ± 37.19 ^c^	580.93 ± 77.86 ^b^
linalool	5.65 ± 0.99 ^a^	5.61 ± 0.15 ^a^	12.00 ± 0.58 ^b^
α-terpineol	3.10 ± 0.37 ^a^	2.94 ± 0.84 ^a^	3.74 ± 0.26 ^a^
β-damascenone	72.97 ± 2.20 ^b^	25.79 ± 2.80 ^a^	23.12 ± 1.74 ^a^
*trans*-geraniol	10.51 ± 2.69 ^a^	16.07 ± 2.02 ^b^	9.77 ± 2.59 ^a^
geranial	40.27 ± 6.03 ^b^	132.88 ± 8.47 ^c^	15.88 ± 1.14 ^a^
nerolidol	20.91 ± 3.43 ^a^	29.97 ± 0.26 ^a^	42.17 ± 7.38 ^b^
6,7-dehydro-7,8-dihydro-3-oxo-ionol	33.44 ± 4.05 ^b^	23.53 ± 0.22 ^a^	28.24 ± 2.09 ^a,b^
3-oxo-α-ionol	102.25 ± 2.09 ^a^	100.39 ± 26.65 ^a^	60.97 ± 12.63 ^a^
Σ Terpenes and norisoprenoids	289.11 ± 2.58 ^b^	337.18 ± 19.73 ^c^	195.89 ± 8.72 ^a^
benzaldehyde	6.53 ± 1.20 ^a^	5.40 ± 0.21 ^a^	6.31 ± 1.52 ^a^
guaiacol	45.59 ± 13.28 ^b^	25.75 ± 3.10 ^a^	54.59 ± 5.01 ^b^
benzyl alcohol	184.07 ± 46.40 ^b^	105.67 ± 3.38 ^a^	214.91 ± 8.77 ^b^
vinylguaicol	72.25 ± 13.59 ^b^	27.08 ± 3.73 ^a^	64.14 ± 5.35 ^b^
syringol	170.76 ± 32.34 ^a^	222.23 ± 23.22 ^a^	187.71 ± 12.31 ^a^
vanillin	7.71 ± 1.66 ^a^	6.31 ± 1.15 ª	6.27 ± 0.31 ª
methyl vanillate	77.23 ± 17.87 ^b^	37.60 ± 2.36 ª	44.89 ± 5.73 ^a^
ethyl vanillate	3.15 ± 0.11 ^a^	24.54 ± 0.81 ^b^	95.49 ± 4.11 ^c^
methyl vanillyl ether	76.73 ± 5.23 ^a^	79.13 ± 3.44 ^a^	84.09 ± 15.05 ^a^
Σ Benzenic compounds	644.00 ± 99.16 ^a,b^	533.72 ± 27.22 ^a^	758.40 ± 29.56 ^b^

Values with different superscripts in the same row denoted significant differences according to the Student–Newman–Keuls test at *p* < 0.05. Samples were defined according to the treatment applied: “Control wine”: no treatment applied; “MW wine”: microwave treatment at 700 W, 12 min of grape crushed; “MW wine without SO_2_”: microwave treatment at 700 W, 12 min of grape crushed elaborated without SO_2_.

**Table 2:** Glycosidically bound volatile compound concentrations (μg/L) in control wines and those obtained from microwave-treated grapes elaborated with and without SO_2_ (*n* = 3).

Volatile Compounds	Control Wine	MW Wine	MW Wine without SO_2_
	Mean ± SD	Mean ± SD	Mean ± SD
1-hexanol	3.73 ± 0.72 ^a^	5.47 ± 1.33 ^a^	4.43 ± 1.04 ^a^
*trans*-3-hexen-1-ol	0.73 ± 0.10 ^a^	6.04 ± 0.08 ^b^	0.79 ± 0.18 ^a^
*cis*-2-hexen-1-ol	2.10 ± 0.17 ^c^	0.83 ± 0.05 ^a^	1.60 ± 0.07 ^b^
*trans*-2-hexen-1-ol	0.66 ± 0.13 ^b^	0.39 ± 0.01 ^a^	0.56 ± 0.04 ^b^
Σ C6 alcohols	7.22 ± 0.82^a^	12.73 ± 1.25 ^b^	7.38 ± 1.02 ^a^
α-terpineol	0.07 ± 0.01 ^b^	Nd	0.04 ± 0.01 ^a^
β-damascenone	0.58 ± 0.13 ^a^	0.60 ± 0.07 ^a^	1.65 ± 0.26 ^b^
*trans*-geraniol	0.83 ± 0.03 ^a^	1.10 ± 0.09 ^b^	1.41 ± 0.43 ^c^
geranic acid	4.45 ± 0.62 ^a^	5.96 ± 0.21 ^b^	3.82 ± 0.10 ^a^
3-oxo-α-ionol	1.77 ± 0.09 ^a^	4.00 ± 1.13 ^b^	2.27 ± 0.51 ^a^
3-hydroxy-7,8-dihydro-β-ionol	0.80 ± 0.09 ^a^	2.00 ± 0.35 ^b^	1.13 ± 0.02 ^a^
Σ Terpenes and norisoprenoids	8.50 ± 0.81 ^a^	13.65 ± 1.69 ^b^	10.33 ± 1.19 ^c^
benzaldehyde	0.70 ± 0.02 ^b^	0.19 ± 0.03 ^a^	0.63 ± 0.17 ^b^
guaiacol	9.09 ± 1.47 ^b^	0.80 ± 0.09 ^a^	16.20 ± 5.01 ^c^
benzyl alcohol	43.46 ± 2.44 ^c^	15.29 ± 0.73 ^b^	6.70 ± 0.57 ^a^
4-vinylguaicol	15.48 ± 0.26 ^a^	20.46 ± 0.24 ^a^	28.90 ± 4.47 ^b^
syringol	116.54 ± 18.60 ^a^	112.95 ± 17.39 ^a^	165.14 ± 34.44 ^a^
benzoic acid	6.72 ± 0.16 ^a^	7.09 ± 1.02 ^a^	10.80 ± 0.89 ^b^
vanillin	1.77 ± 0.07 ^a^	2.12 ± 0.29 ^b^	1.67 ± 0.02 ^a^
methyl vanillate	6.82 ± 1.12 ^a^	5.53 ± 1.05 ^a^	10.51 ± 0.97 ^b^
ethyl vanillate	nd	14.16 ± 1.41	nd
Σ Benzenic compounds	200.57 ± 19.66 ^a^	178.58 ± 20.84 ^a^	240.56 ± 41.44 ^a^

Values with different superscripts in the same row denoted significant differences according to the Student–Newman–Keuls test at *p* < 0.05. Samples were defined according to the treatment applied: “Control wine”: no treatment applied; “MW wine”: microwave treatment at 700 W, 12 min of grape crushed; “MW wine without SO_2_”: microwave treatment at 700 W, 12 min of grape crushed elaborated without SO_2_. nd: not detected.

**Table 3:** Fermentative volatile compound concentration (μg/L) in control wines and those obtained from microwave-treated grapes and elaborated with and without SO_2_ (*n* = 3).

Volatile Compounds	Control Wine	MW Wine	MW Wine without SO_2_
	Mean ± SD	Mean ± SD	Mean ± SD
2-methyl-1-propanol	38.68 ± 4.74 ^a^	154.91 ± 14.26 ^b^	245.16 ± 9.03 ^c^
butanol	13.16 ± 5.41 ^a,b^	16.17 ± 0.32 ^b^	7.47 ± 0.01 ^a^
3-methyl-1-pentanol	4.13 ± 0.67 ^a^	33.20 ± 6.28 ^c^	15.09 ± 3.61 ^b^
4-methyl-1-pentanol	17.58 ± 0.79 ^a^	102.74 ± 21.09 ^b^	26.80 ± 4.26 ^a^
3-octanol	17.75 ± 3.57 ^b^	1.85 ± 0.48 ^a^	3.69 ± 0.40 ^a^
1-octen-3-ol	2.55 ± 0.15 ^b^	1.66 ± 0.31 ^a^	4.01 ± 0.52 ^c^
1-heptanol	2.12 ± 0.39 ^a^	25.25 ± 0.45 ^c^	11.90 ± 3.24 ^b^
1-octanol	1.41 ± 0.18 ^a^	4.30 ± 0.19 ^b^	5.85 ± 1.33 ^b^
Σ Alcohols	97.38 ± 3.12 ^a^	340.07 ± 28.58 ^b^	319.96 ± 2.22 ^b^
isobutyric acid	53.62 ± 7.38 ^a^	64.50 ± 3.78 ª	57.53 ± 5.72 ^a^
butanoic acid	5.87 ± 0.18 ^a^	36.47 ± 1.30 ^c^	13.10 ± 0.31 ^b^
isovaleric acid	435.90 ± 48.07 ^a^	474.04 ± 13.00 ^a^	438.50 ± 25.45 ^a^
pentanoic acid	2.84 ± 0.44 ^a^	19.65 ± 1.81 ^b^	3.26 ± 0.14 ^a^
octanoic acid	1020.51 ± 19.23 ^b^	1296.23 ± 48.52 ^c^	621.99 ± 12.47 ^a^
dodecanoic acid	57.93 ± 6.19 ª	66.54 ± 11.11 ª	78.01 ± 5.93 ^a^
Σ Acids	1576.66 ± 74.02 ^b^	1957.43 ± 56.69 ^c^	1212.39 ± 20.09 ^a^
ethyl butyrate	12.62 ± 1.09 ^a^	61.87 ± 1.02 ^c^	40.84 ± 0.77 ^b^
ethyl isovalerate	4.14 ± 0.43 ^a^	10.32 ± 2.44 ^b^	15.00 ± 0.50 ^c^
isoamyl acetate	1437.24 ± 53.61 ^a^	2066.95 ± 41.66 ^b^	946.51 ± 12.57 ^a^
ethyl hexanoate	217.70 ± 38.39 ^a^	327.35 ± 37.71 ^b^	236.58 ± 15.79 ^a^
ethyl piruvate	3.42 ± 0.20 ^a^	7.76 ± 0.01 ^b^	8.11 ± 1.12 ^b^
hexyl acetate	1.88 ± 0.52 ^a^	18.15 ± 3.29 ^b^	5.25 ± 1.06 ª
ethyl octanoate	262.41 ± 26.43 ^b^	285.79 ± 15.27 ^b^	84.76 ± 20.38 ^a^
ethyl decanoate	22.68 ± 4.74 ^a^	114.82 ± 9.64 ^b^	33.76 ± 5.83 ^a^
ethyl succinate	473.01 ± 5.05 ^b^	443.08 ± 15.26 ^b^	273.41 ± 73.23 ^a^
ethyl 2-(OH)-4-methylpentanoate	41.38 ± 1.29 ^a^	32.22 ± 0.98 ^a^	36.59 ± 9.42 ^a^
ethyl 3-(OH)-hexanoate	7.74 ± 0.31 ^b^	7.45 ± 1.21 ^b^	1.40 ± 0.38 ^a^
ethyl 4-(OH)-butanoate	6594.31 ± 155.03 ^c^	3116.845 ± 234.87 ^b^	1991.30 ± 412.43 ^a^
Σ Esters	9078.54 ± 217.17 ^c^	6492.62 ± 222.50 ^b^	3673.50 ± 482.15 ^a^
2-phenylethyl acetate	76.36 ± 7.55 ^a^	425.68 ± 15.83 ^c^	175.51 ± 47.70 ^b^
2-phenylethanol	24303.25 ± 555.69 ^a^	35620.5 ± 2701.47 ^b^	43608.55 ± 1702.30 ^e^
4-vinyl-phenol	311.78 ± 24.28 ^a^	417.51 ± 15.50 ^b^	431.89 ± 39.91 ^b^
tyrosol	3485.19 ± 839.37 ^c^	2411.07 ± 7.49 ^b^	1284.24 ± 374.96 ^a^
benzeneacetic acid	10.19 ± 2.34 ^a^	24.20 ± 0.81 ^b^	39.38 ± 5.46 ^c^
(2-phenylethyl) acetamide	56.51 ± 7.36 ^a^	49.32 ± 5.69 ^a^	99.23 ± 15.67 ^b^
Σ Benzenic compounds	28243.26 ± 1370.95 ^a^	38948.27 ± 2684.29 ^b^	45638.81 ± 1402.11 ^c^
3-(methylthio)-1-propanol	105.56 ± 13.62 ^c^	38.72 ± 4.69 ^a^	78.50 ± 10.71 ^b^
3-(2H)-thiophenone, dihydro-2-methyl	204.19 ± 15.73 ^c^	136.46 ± 2.75 ^b^	50.87 ± 7.30 ^a^
γ-butyrolactone	646.20 ± 50.68 ^b^	49.33 ± 14.33 ^a^	27.55 ± 4.40 ^a^
γ-nonalactone	10.28 ± 1.35 ^a^	15.29 ± 2.23 ª	15.83 ± 3.99 ª
pantolactone	13.26 ± 1.63 ^b^	6.87 ± 2.36 ^a^	14.09 ± 2.40 ^b^
γ-decalactone	0.43 ± 0.03 ^a^	2.05 ± 0.07 ^c^	0.84 ± 0.13 ^b^
Σ Furans & sulfur compounds	979.92 ± 60.10 ^b^	248.72 ± 13.59 ^a^	187.69 ± 9.18 ^a^

Values with different superscripts in the same row denoted significant differences according to the Student–Newman–Keuls test at *p* < 0.05. Samples were defined according to the treatment applied: “Control wine”: no treatment applied, 7 days of maceration; “MW wine”: microwave treatment at 700 W, 12 min of grape crushed, 7 d of maceration; “MW wine without SO_2_”: microwave treatment at 700 W, 12 min of grape crushed, 7 d of maceration, elaborated without SO_2_.

**Table 4:** Odor-active values of the most sensorially significant volatile compounds in control wines and microwave-treated wines fermented with and without SO_2_.

Volatile Compound	Odor Threshold μg/L *	Odor Descriptor	OAV **		
			Control Wine	MW Wine	MW Wine without SO_2_
linalool	15	Citrus, floral, sweet	0.4	0.4	0.8
β-damascenone	0.05	Honey, sweet,	1459.4	515.8	462.4
guaiacol	10	Smoke, sweet, medicine	4.6	2.6	5.5
vinylguaicol	40	Spices, curry	1.8	0.7	1.6
ethyl butyrate	20	Fruity, strawberry, sweet,	0.6	3.1	2.0
ethyl isovalerate	3	Apple, sweet	1.4	3.4	5.0
isoamyl acetate	30	Banana, fruity, sweet	47.9	68.9	31.5
ethyl hexanoate	5	Fruity, green, apple.	43.5	65.5	47.3
ethyl octanoate	5	Sweet, fruity, pear	52.5	57.2	16.9
2-phenylethyl acetate	250	Flowery	0.3	1.7	0.7
2-phenylethanol	10,000	Rose, honey	2.4	3.5	4.3
γ-nonalactone	25	soft coconut, sweet	0.4	0.6	0.6

* odor thresholds values have been obtained from the References: [33,38]. ** OAVs were calculated dividing the mean concentration of each compound in wines by its odor threshold.

## Data Availability

Not available.

## Supplementary Materials

The following are available online: https://www.mdpi.com/article/10.3390/foods10061164/s1 Table S1. Free volatile compound concentrations (μg/L) in control must and that obtained from microwave-treated grapes (MW). Table S2. Glycosidically bound volatile compound concentrations (μg/L) in control must (C) and that obtained from microwave-treated grapes (MW).
