# Supplementary material for: Effect of Microwave Maceration and SO2 Free Vinification on Volatile Composition of Red Wines
Source: Foods. 2021 May 22;10(6):1164. doi: 10.3390/foods10061164 (PMC8224603; doi:10.3390/foods10061164)
Supplement: Supplementary file 1 [file foods-10-01164-s001.zip › foods-1210763-supplementary.pdf]

Supplementary Table S1. Free volatile compound concentration (μg/L) in control must and that obtained from microwave-treated grapes (MW).

Volatile compounds	C	MW
	Mean ± SD	Mean ± SD
hexanal*	246.72 ± 7.81	59.66 ± 1.90
trans-2-hexenal*	79.30 ± 1.53	10.98 ± 1.52
octanal	5.08 ± 1.02	0.89 ± 0.01
1-hexanol*	86.78 ± 0.54	273.45 ± 0.75
cis-3-hexen-1-ol*	6.12 ± 0.08	19.31 ± 0.20
trans-3-hexen-1-ol*	17.53 ± 0.01	47.86 ± 1.12
nonanal	7.98 ± 0.01	6.98 ± 0.62
cis-2-hexen-1-ol*	256.22 ± 3.89	924.48 ± 3.20
trans-2-hexen-1-ol	1.66 ± 0.20	7.55 ± 0.09
benzaldehyde	0.78 ± 0.08	0.82 ± 0.11
β-damascenone	nd	0.14 ± 0.03
trans-geraniol*	0.88 ± 0.09	3.18 ± 0.08
guaiacol	0.91 ± 0.27	3.31 ± 0.69
benzylalcohol*	51.21 ± 3.44	106.18 ± 2.87
2-phenylethanol*	15.56 ± 0.01	45.97 ± 1.88
4-vinyl-guaiacol*	9.50 ± 0.31	14.29 ± 3.19
syringol	4.92 ± 0.09	7.32 ± 4.10
vanillin*	13.30 ± 0.98	23.59 ± 2.50
methyl vanillate*	nd	7.84 ± 0.49
acetovanillone*	nd	55.39 ± 68.51

*: denote significant differences between means data according to de *t*-Student test (Significance $p \leq 0.05$). nd: not detected.

Supplementary Table S2. Glycosidically-bound volatile compound concentrations (μg/L) in control must (C) and that obtained from microwave-treated grapes (MW).

Volatile compounds	C	MW
	Mean ± SD	Mean ± SD
hexanal	7.23 ± 0.02	6.62 ± 0.19
trans-2-hexenal	17.68 ± 0.06	6.76 ± 0.25
octanal	23.88 ± 3.42	18.63 ± 0.88
1-hexanol*	0.65 ± 0.02	5.42 ± 0.01
trans-3-hexen-1-ol*	nd	1.01 ± 0.01
nonanal*	0.65 ± 0.00	1.31 ± 0.06
cis-2-hexen-1-ol*	0.41 ± 0.01	2.13 ± 0.04
trans-2-hexen-1-ol	nd	0.05 ± 0.03
benzaldehyde	1.90 ± 0.24	4.55 ± 0.11
β-damascenone	3.57 ± 0.05	3.86 ± 0.12
trans-geraniol	3.89 ± 0.01	3.83 ± 0.60
guaiacol	0.72 ± 0.02	2.74 ± 0.80
benzylalcohol*	nd	65.51 ± 5.41
2-phenylethanol*	6.25 ± 0.59	62.92 ± 0.62
4-vinyl-guaiacol*	29.99 ± 5.46	150.76 ± 62.64
syringol	4.97 ± 0.29	14.09 ± 10.04
3-hydroxy-β-damascone*	2.94 ± 0.09	12.26 ± 1.35
vanillin*	3.26 ± 0.01	5.51 ± 0.12
methyl vanillate*	6.57 ± 1.01	26.83 ± 2.92
acetovanillone*	3.34 ± 1.05	24.74 ± 0.82

*: denote significant differences between means data according to de *t*-Student test (Significance $p \leq 0.05$). nd: not detected.
